# Genome-wide association study for renal traits in the Framingham Heart and Atherosclerosis Risk in Communities Studies

**DOI:** 10.1186/1471-2350-9-49

**Published:** 2008-06-03

**Authors:** Anna Kottgen, Wen Hong L Kao, Shih-Jen Hwang, Eric Boerwinkle, Qiong Yang, Daniel Levy, Emelia J Benjamin, Martin G Larson, Brad C Astor, Josef Coresh, Caroline S Fox

**Affiliations:** 1 Department of Epidemiology and Welch Center for Prevention, Epidemiology & Clinical Research, Johns Hopkins University, Baltimore, MD, USA; 2 Department of Medicine, School of Medicine, Johns Hopkins University, Baltimore, MD, USA; 3 Center for Population Studies, NHLBI, Bethesda, MD and NHLBI's Framingham Heart Study, Framingham, MA, USA; 4 Human Genetics Center and Institute of Molecular Medicine, University of Texas Health Science Center, Houston, TX, USA; 5 Department of Biostatistics, Boston University School of Public Health, Boston, MA, USA; 6 Division of Cardiology and Department of Preventive Medicine, School of Medicine, Department of Epidemiology, School of Public Health, Boston University, Boston, MA, USA; 7 Department of Biostatistics, Johns Hopkins Bloomberg School of Public Health, Baltimore, MD, USA; 8 Division of Endocrinology, Hypertension, and Metabolism, Brigham and Women's Hospital and Harvard Medical School, Boston, MA, USA

## Abstract

**Background:**

The Framingham Heart Study (FHS) recently obtained initial results from the first genome-wide association scan for renal traits. The study of 70,987 single nucleotide polymorphisms (SNPs) in 1,010 FHS participants provides a list of SNPs showing the strongest associations with renal traits which need to be verified in independent study samples.

**Methods:**

Sixteen SNPs were selected for replication based on the most promising associations with chronic kidney disease (CKD), estimated glomerular filtration rate (eGFR), and serum cystatin C in FHS. These SNPs were genotyped in 15,747 participants of the Atherosclerosis in Communities (ARIC) Study and evaluated for association using multivariable adjusted regression analyses. Primary outcomes in ARIC were CKD and eGFR. Secondary prospective analyses were conducted for association with kidney disease progression using multivariable adjusted Cox proportional hazards regression. The definition of the outcomes, all covariates, and the use of an additive genetic model was consistent with the original analyses in FHS.

**Results:**

The intronic SNP rs6495446 in the gene *MTHFS *was significantly associated with CKD among white ARIC participants at visit 4: the odds ratio per each C allele was 1.24 (95% CI 1.09–1.41, p = 0.001). Borderline significant associations of rs6495446 were observed with CKD at study visit 1 (p = 0.024), eGFR at study visits 1 (p = 0.073) and 4 (lower mean eGFR per C allele by 0.6 ml/min/1.73 m^2^, p = 0.043) and kidney disease progression (hazard ratio 1.13 per each C allele, 95% CI 1.00–1.26, p = 0.041). Another SNP, rs3779748 in *EYA1*, was significantly associated with CKD at ARIC visit 1 (odds ratio per each T allele 1.22, p = 0.01), but only with eGFR and cystatin C in FHS.

**Conclusion:**

This genome-wide association study provides unbiased information implicating *MTHFS *as a candidate gene for kidney disease. Our findings highlight the importance of replication to identify common SNPs associated with renal traits.

## Background

Kidney disease aggregates within families and measures of kidney function, such as estimated glomerular filtration rate (eGFR), are heritable [1-4]. Whereas many monogenetic causes of kidney disease have been discovered, the identification of common genetic variants hypothesized to confer susceptibility to complex diseases, such as chronic kidney disease (CKD), has been difficult and suffers from a lack of replication of initial positive findings [1,5].

Recently, genome-wide association studies (GWAS) to discover associations of common genetic variants, single nucleotide polymorphisms (SNPs), and a phenotype of interest have become feasible. Surveying the whole genomes of many individuals, preferably as part of a large prospective study that provides extensive and rigorously collected information on phenotypes, can provide unbiased findings and has the power to potentially discover common genetic variants that are associated with complex diseases. So far, the method has successfully and repeatedly identified common SNPs associated with a wide variety of complex diseases such as diabetes mellitus and coronary heart disease [6-11]. Because of the large number of tests conducted and the small *a priori *probability of a true association between any given SNP and the phenotype, replication of initial findings from a GWAS is essential [12].

Recently, genome-wide tests of 70,987 autosomal SNPs with renal traits were conducted as part of the Framingham Heart Study (FHS) 100 K SNP GWAS resource [13]. Initial results have been published [14], but have not been replicated in independent study samples to date. None of the initial associations between SNPs and CKD or eGFR reached genome-wide significance. However, the initial study had limited power, and true associations of moderate size are likely to be associated at p-values on the order of 10e-3 to 10e-5, as will be false positive associations due to the large number of tests conducted. To distinguish true from false positive findings, promising SNPs need to be tested in independent cohorts. Therefore, the objective of the present study was to validate initial findings by replicating the strongest and most promising associations after correction for multiple testing. Here we report the first replication of initially observed associations from a GWAS of kidney disease traits in 11,447 white participants of the community-based, prospective Atherosclerosis Risk in Communities (ARIC) Study.

## Methods

### Description of the initial (stage I) sample, Framingham Heart Study

#### Study sample

In 1948, 5,209 participants of the Framingham Heart Study, a prospective community-based cohort, were recruited into the Original Cohort. In 1971, 5,124 of their children or spouses were enrolled into the Offspring Cohort and examined every 4 to 8 years [15]. Members of the largest 330 pedigrees among the Original and Offspring Cohorts were selected for genotyping (n = 1,345 after data cleaning) as detailed elsewhere [13]. Of these, phenotype data from the Offspring examination 7 in 1998–2001 were available for 1,010 individuals with eGFR and CKD and 981 individuals with cystatin C measurements, the final sample sizes used for the stage I analyses of the GWAS of renal traits [14]. The study was approved by the Institutional Review Board of the Boston University Medical Center. All subjects provided written informed consent.

#### Genotyping

Genotyping was performed using the Affymetrix GeneChip Human Mapping 100 K SNP set. Details of the genotyping process are reported elsewhere [13]. All genotype data were returned to the NHLBI; aggregate results data are publicly available [16]. SNPs with call rates <80%, deviations from Hardy-Weinberg expectations (p < 0.001 in unrelated individuals) or minor allele frequency <10% were excluded. After data cleaning, 70,987 autosomal SNPs remained for analyses [14].

#### Outcome definition

Estimated GFR (ml/min/1.73 m^2^) was calculated using the four-variable Modification of Diet in Renal Disease Study equation[17] from calibrated serum creatinine measured at Offspring examination 7 by the modified Jaffe method. CKD was defined based on the National Kidney Foundation Kidney Disease Outcome Quality Initiative working group [18], and modified slightly, using sex-specific cutoffs for CKD of eGFR<59 ml/min/1.73 m^2 ^in women and <64 ml/min/1.73 m^2 ^in men, as described previously [14,19]. Serum cystatin C (mg/l) was measured at Offspring examination 7 using particle enhanced immunonephelometry (Dade Behring BN 100 nephelometer) [14].

#### Statistical analysis

For data analysis, multivariable-adjusted residuals were generated for each phenotype. Covariates used for multivariable-adjustment were age, sex, systolic blood pressure, hypertension treatment, HDL-cholesterol, smoking, diabetes, and body mass index [20]. To account for relatedness among the study individuals, generalized estimating equations (GEE) or family-based association tests (FBAT) were used to test associations between phenotype residuals and each SNP; a detailed description is provided elsewhere [13]. Additional analyses re-analyzed SNPs that replicated in ARIC using the raw traits in multivariable adjusted GEE regression models to allow for a direct comparison of effect size estimates between the two studies.

#### Selection of SNPs for replication

**Figure 1:**
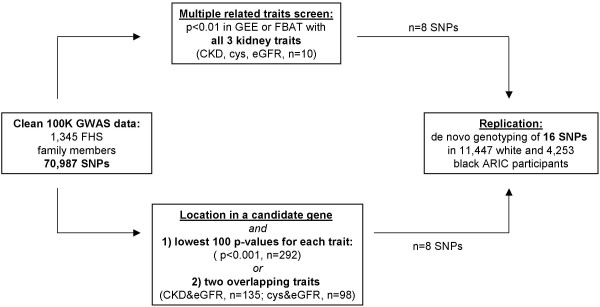
**Selection process of SNPs from the FHS 100 K GWAS screen to be followed up by genotyping in the ARIC cohort**. Abbreviations: GWAS: genome-wide association, SNPs: single nucleotide polymorphisms, GEE: generalized estimating equation, FBAT: family-based association test, CKD: chronic kidney disease, eGFR: estimated glomerular filtration rate, cys: cystatin C.

Strongly associated SNPs in the FHS 100 K analyses were prioritized for follow-up genotyping in an independent sample, the ARIC Study (described below) as shown in Figure 1. Ten SNPs showed joint association with all three kidney traits (eGFR, CKD, cystatin C) at p < 0.01 for each trait. Eight of these 10 SNPs were selected for follow-up genotyping; the remaining 2 were dropped due to high linkage disequilibrium (LD) with other SNPs in this set. In addition, statistical and biological evidence were combined by selecting a subset of SNPs showing strong association and additional location in candidate gene regions. Eight SNPs met these criteria, and were selected from the following sets: 1) SNPs with the 100 lowest p-values from GEE models for association with one of the 3 kidney traits; 2) SNPs that showed association with both eGFR and cystatin C at p-values <0.01 from either GEE or FBAT models; 3) SNPs that showed association with both eGFR and CKD at p-values <0.01 from either GEE or FBAT models. Only SNPs with a minimum call rate of 90% were considered for replication. Candidate genes were identified as auch if, based on a thorough literature search, there was evidence for the gene's involvement in renal disease (for example, a renal phenotype in knock-out mice or mutations causing a Mendelian syndrome in humans with renal involvement), or if their gene products are known to be involved in a physiological mechanism important for kidney function such as filtration or electrolyte transport. The SNPs selected for replication for their location in or near a candidate gene were located in *FRAS1*,[21]*NR3C2*,[22]*SGK1*,[23]*CFTR*,[24]*EYA1*,[25]*IQGAP1*,[26,27] and near *GLIS3*[28].

### Description of the replication (stage II) sample, Atherosclerosis in Communities Study

#### Study sample

The ARIC Study is an ongoing, population-based, prospective cohort of 15,792 adults. Study participants were aged 45–64 years at their recruitment from four US communities in 1987–89, when they underwent the standardized ARIC baseline examination (visit 1). Participants underwent 3 further standardized examinations approximately every 3 years. Since the end of visit 4 in 1998, participants continue to be followed up by annual phone calls as well as by obtaining information from hospitalization discharge records of local hospitals and death certificates. Further details of the study design have been reported previously [29]. Institutional Review Boards of the participating institutions approved the study protocols, and written informed consent was obtained from all participants at each examination. From the total study sample of 15,792 participants at the baseline examination, 45 individuals were excluded because they did not consent to genetic research and 47 because they did not self-identify as "black" or "white". As the genotyped FHS participants were exclusively white, the primary replication sample consisted of white ARIC participants. Black ARIC participants were also genotyped, enabling further characterization of SNPs showing evidence of association in white ARIC participants. Measures of genotyping quality control and allele distributions were assessed in 11,447 white and 4,253 black ARIC participants. For association analyses, participants were additionally excluded for genotyping failure of all SNPs (173 whites, 168 blacks), and for missing serum creatinine (4 whites, 97 blacks) and missing covariates (53 whites, 94 blacks) at visit 1 [missing creatinine (76 whites, 77 blacks) and missing covariates (15 whites, 135 blacks) at visit 4] for a final, primary replication study sample of 11,217 white participants at visit 1 [8,717 at visit 4] (Table 1), and additionally 3,894 black participants at visit 1 [2,358 at visit 4] [see Additional file 1].

**Table 1:** Study Characteristics by Study Sample

	**Framingham Heart Study**	**ARIC whites**	
	**Examination 7**	**Visit 1**	**Visit 4**

Characteristic			
Sample size n	1007	11217	8717
Age, years	58.5 (9.6)	54.4 (5.7)	63.1 (5.6)
Male, %	49.1	47.2	46.3
Systolic blood pressure, mmHg	125 (18.5)	118.5 (17)	125.5 (18)
Antihypertensive medication, %	31.2	25.8	31.4
Diabetes mellitus, %	11.2	9.1	13.9
Body mass index, kg/m^2^	28.4 (5.8)	27.0 (4.9)	28.3 (5.2)
Current smokers, %	14.2	24.8	14.1
High density lipoprotein cholesterol, mg/dl	52.6 (16.1)	50.4 (16.8)	49.0 (16.3)
Serum creatinine, mg/dl	0.88 (0.28)	0.85 (0.20)	0.91 (0.24)
eGFR, ml/min/1.73 m^2^	86.6 (19.8)	89.6 (17.9)	80.5 (16.9)
Prevalent CKD*, n (%)	80 (8.0)	449 (4.0)	756 (8.7)
Incident CKD^†^, n	N/A	N/A	843
Serum cystatin C, mg/l	0.96 (0.24)	N/A	N/A

Data are presented as mean (standard deviation) for continuous and proportion for categorical variables for individuals with information on all characteristics available at FHS Offspring exam 7 and ARIC visits 1 and 4. *CKD was defined as eGFR <59 ml/min/1.73 m^2 ^(women) and <64 ml/min/1.73 m^2 ^(men). ^†^For definition of incident CKD and duration of follow-up see methods section. Abbreviations: eGFR: estimated glomerular filtration rate, CKD: chronic kidney disease.

#### Assessment of other study characteristics

Detailed information on obtaining demographic, socioeconomic, health behavior, risk factor control, and medical history have been described previously [29,30]. Racial affiliation was self-reported using the terms "black" or "white".

#### Genotyping

Genotyping of all SNPs was performed by the ARIC Central DNA Laboratory. The polymorphisms rs10509132, rs2827732, rs6495446, rs1743955, and rs4835136 were genotyped individually using the TaqMan assay (Applied Biosystems, Foster City, CA). All other SNPs were genotyped in two multiplexes using the iPLEX gold assay (Sequenom, San Diego, CA). The minimum satisfactory call rate was set at 90%, and the cutoff indicating statistically significant deviation from Hardy-Weinberg expectations was set at p < 10^-4^.

#### Outcome definitions

Continuous eGFR and CKD, defined as in FHS, were defined *a priori *as the primary study outcomes. These outcomes were investigated primarily at ARIC visit 4 which provided more individuals with CKD and a distribution of major kidney disease risk factors, such as hypertension, similar to that in FHS. Outcomes were also investigated at ARIC visit 1 to maximize the sample size for continuous outcomes. Serum cystatin C measurements were not available in the ARIC Study. In secondary analyses, associations between the SNPs and kidney disease progression were conducted, as were analyses defining CKD as eGFR <60 ml/min/1.73 m^2 ^[18]. Serum creatinine was measured using the modified kinetic Jaffe reaction and creatinine values were standardized and calibrated as described previously [31,32]. Kidney disease progression was defined as either an increase in serum creatinine levels ≥ 0.4 mg/dl above baseline or a hospitalization discharge or death coded for chronic renal disease using international classification of disease codes and analyzed as described elsewhere [30].

#### Statistical analysis

Consistent with FHS, an additive genetic model was used for each SNP in all analyses. Multivariable regression models were adjusted for the same covariates as in FHS. For the secondary analyses using prospective data, follow-up time was counted from the baseline ARIC visit until the date kidney disease progression occurred in cases, or the earlier of the date of last contact (or date of non-CKD death) or December 31, 2004 for non-cases. Multivariable adjusted Cox Proportional Hazards models were used, and the proportionality assumption of all Cox models was assessed by inspection of the complementary log(-log [survival function]) curves.

The two-sided significance level was determined a *priori *at α = 0.05 for each of the 8 SNPs in plausible candidate genes and at α = 0.00625 (0.05/8) after applying a Bonferroni correction to the other 8 SNPs. In our analyses among white participants, the power to detect an odds ratio of CKD of 1.3 or higher per each increase in risk allele and assuming α = 0.05 is >80% for a risk allele frequency of 0.2, and >90% for a risk allele frequency of 0.3.

## Results

Table 1 shows the distribution of study sample characteristics among FHS and white ARIC participants, and Additional file 1 shows this distribution among the additional study sample of black ARIC participants. The mean eGFR in FHS participants was 86.6 ml/min/1.73 m^2 ^(standard deviation (SD) 19.8), compared to 89.6 ml/min/1.73 m^2 ^(SD 17.9) in white ARIC participants at visit 1 and 80.5 ml/min/1.73 m^2 ^(SD 16.9) at visit 4. CKD was present in 80 (7.9%) of FHS participants and in 449 (4.0%) of white ARIC participants at visit 1 and 756 (8.7%) at visit 4.

Minor allele frequencies for the 16 SNPs among FHS and white ARIC participants are provided in Table 2, and minor allele frequencies among black ARIC participants are provided separately [see Additional file 2]. The minor allele frequencies in FHS and white ARIC participants were similar, and both were similar to the minor allele frequencies from the HapMap CEU sample. The distribution of genotypes for all SNPs in ARIC whites conformed to Hardy-Weinberg expectations, while in ARIC blacks, rs2228210 and rs6831700 did not and were excluded from further analyses. The average call rate across SNPs was 94.1% in white and 91.6% in black ARIC participants. The concordance rates for genotyping of 314 replicate samples per SNP in ARIC was >97% for each SNP (kappa coefficient ≥ 0.95).

**Table 2:** Minor allele frequencies for all SNPs genotyped in 1,345 FHS and 11,447 white ARIC participants

			**n**		**MAF**	
**SNP**	**gene**	**location**	**FHS**	**ARIC white**	**FHS**	**ARIC white**

***Selected for low p-value only***						
rs4553158	*MIER1*	chr1:67148467	1,219	10,685	0.13 (G)	0.17 (G)
rs6831700	*WDR19*	chr4:39079530	1,273	10,808	0.34 (G)	0.33 (G)
rs2419912	(BC047601)	chr5:157777113	1,341	10,817	0.49 (C)	0.46 (C)
rs2228210	*HIVEP1*	chr6:12230160	1,252	10,782	0.29 (G)	0.36 (G)
rs10509132	*ANK3*	chr10:61995671	1,223	10,783	0.45 (G)	0.48 (G)
rs1613631	*KRT84*	chr12:51062202	1,330	10,782	0.20 (G)	0.20 (G)
rs6495446	*MTHFS*	chr15:77942037	1,328	10,816	0.24 (T)	0.27 (T)
rs2827732	gene desert	chr21:23107838	1,344	10,768	0.19 (A)	0.16 (A)
						
***Selected as a candidate***						
rs2061063	*FRAS1*	chr4:79591766	1,322	10,747	0.33 (G)	0.35 (G)
rs4835136	*NR3C2*	chr4:149627601	1,334	10,990	0.36 (T)	0.37 (T)
rs1743955	*SGK1*	chr6:134562589	1,299	10,763	0.42 (T)	0.39 (T)
rs4148686	*CFTR*	chr7:116728468	1,225	10,795	0.17 (G)	0.20 (G)
rs3779748	*EYA1*	chr8:72410728	1,342	10,789	0.32 (C)	0.33 (C)
rs1455177	BC047388	chr9:3782613	1,337	10,434	0.45 (G)	0.45 (G)
rs10520688	*IQGAP1*	chr15:88752520	1,331	10,814	0.15 (G)	0.14 (G)
rs2839235	*PCNT*	chr21:46625020	1,300	10,799	0.14 (C)	0.13 (C)

To facilitate interpretation, alleles were transferred to the strand typed in ARIC if they were typed on the opposite strand in FHS. The sample size n presented here is for all successfully genotyped individuals irrespective of whether all covariates for regression analyses were available. Abbreviations: SNPs: single nucleotide polymorphisms, n: sample size, MAF: minor allele frequency.

### Associations of SNPs with eGFR and CKD in the ARIC Study

Table 3 shows the replication data for the 16 selected SNPs at visit 4, where participants were on average 9 years older than at visit 1, thus providing a greater number of participants with CKD. Among the 8 SNPs associated with all 3 renal traits in FHS, rs6495446 in *MTHFS *was significantly associated with CKD among white ARIC participants (odds ratio (OR) 1.24 per each C allele, 95% confidence interval 1.09–1.41, p = 0.001). The association with eGFR at visit 4 (-0.57 ml/min/1.73 m^2 ^per increase in C allele, p = 0.043) was not significant at the pre-specified α of 0.00625. The proportion of eGFR variance explained by rs6495446 was 0.04% (0.4% in FHS). The associations of rs6495446 with CKD and eGFR were of similar magnitude but not statistically significant at ARIC visit 1 (OR of CKD 1.20 per increase in C allele, p = 0.024, -0.48 ml/min/1.73 m^2 ^per increase in C allele, p = 0.073) [see Additional file 3]. The direction of the association results for rs6495446 was consistent with the one observed in FHS, therefore replicating the association of this SNP with CKD. Further investigation of this SNP in black ARIC participants revealed no significant association of rs6495446 and either trait at visit 4 (p = 0.68 for CKD and p = 0.26 for eGFR) or visit 1 (p = 0.44 for CKD and p = 0.03 for eGFR), although the association of rs6495446 and eGFR at visit 1 in ARIC blacks was of borderline significance (-1.2 ml/min/1.73 m^2 ^per increase in T allele, p = 0.03). None of the other 7 SNPs associated with all 3 renal traits in FHS were significantly associated with either kidney trait in white ARIC participants at visit 4 (Table 3).

**Table 3:** Replication data of 16 SNPs significantly associated with renal traits on the FHS 100 K chip in 8,717 white participants at ARIC visit 4.

**SNP**	**Gene**	**Strand**	Alleles*	**MAF**		**FHS**				**ARIC**
		**FHS/ARIC**		**FHS**	**ARIC**	**Trait List†**	**Trait**	**GEE p-value**	**FBAT p-value**	**GLM p-value**

***Selected for low p-value only***										
rs4553158	*MIER1*	-/+	A/G	0.13(C)	0.17(G)	1,2,3,4	CKD	4.3xe^-4^	0.739	0.749
							cys	0.006	0.432	--
							eGFR	0.003	0.914	0.300
rs6831700	*WDR19*	-/+	G/T	0.34(C)	0.33(G)	1	CKD	0.004	0.001	0.361
							cys	0.344	0.005	--
							eGFR	0.034	0.001	0.472
rs2419912	(BC047601)	+/+	T/C	0.49(C)	0.46(C)	1	CKD	0.002	0.144	0.044
							cys	0.003	0.030	--
							eGFR	9.4xe^-5^	0.342	0.026
rs2228210	*HIVEP1*	+/+	A/G	0.29(G)	0.36(G)	1,2,3	CKD	0.017	0.007	0.805
ns cSNP							cys	0.003	0.027	--
							eGFR	2.3xe^-4^	0.005	0.520
rs10509132	*ANK3*	+/+	G/T	0.45(G)	0.48(G)	1,2,3	CKD	0.009	0.855	0.609
							cys	0.002	0.002	--
							eGFR	0.002	0.031	0.349
rs1613631	*KRT84*	-/+	T/G	0.2(C)	0.20(G)	1,2,3	CKD	0.048	0.002	0.360
							cys	0.177	0.009	--
							eGFR	0.013	0.003	0.169
rs6495446	*MTHFS*	+/+	C/T	0.24(T)	0.27(T)	1,2,3	CKD	0.003	0.429	0.001
							cys	0.006	0.149	--
							eGFR	0.001	0.167	0.043
rs2827732	gene desert	-/+	C/A	0.19(T)	0.16(A)	1,2,3	CKD	0.001	0.004	0.403
							cys	0.002	4.9 × 10^-4^	--
							eGFR	0.002	0.025	0.806
***Selected as a candidate***										
rs2061063	*FRAS1*	-/-	G/C	0.33(G)	0.35(G)	2	CKD	0.009	0.015	0.149
							cys	0.402	0.790	--
							eGFR	0.009	0.018	0.719
rs4835136	*NR3C2*	-/+	C/T	0.36(A)	0.37(T)	2,5	CKD	0.003	0.002	0.380
							cys	0.971	0.260	--
							eGFR	0.001	0.001	0.920
rs1743955	*SGK1*	-/+	T/C	0.42(A)	0.39(T)	3	CKD	0.707	0.459	0.164
							cys	0.096	0.004	--
							eGFR	0.006	0.007	0.889
rs4148686	*CFTR*	-/+	C/G	0.17(C)	0.20(G)	4	CKD	1.1xe^-4^	0.209	0.543
							cys	0.914	0.291	--
							eGFR	0.060	0.555	0.353
rs3779748	*EYA1*	+/+	T/C	0.32(C)	0.33(C)	3	CKD	0.128	0.826	0.882
							cys	0.008	0.435	--
							eGFR	0.006	0.593	0.788
rs1455177	BC047388	+/-	C/G	0.45(C)	0.45(G)	6	CKD	0.113	0.494	0.208
	(GLIS3)						cys	6.0xe^-4^	0.045	--
							eGFR	0.059	0.669	0.404
rs10520688	*IQGAP1*	+/-	T/C	0.15(C)	0.14(G)	3	CKD	0.043	0.837	0.020
							cys	0.004	0.608	--
							eGFR	0.003	0.82	0.674
rs2839235	*PCNT*	+/+	T/C	0.14(C)	0.13(C)	3,5	CKD	0.028	0.134	0.848
							cys	0.016	0.006	--
							eGFR	1.6xe^-5^	0.055	0.827

The upper part of the table contains the SNPs selected for replication based on a low p-value (<0.01) for association with all of the 3 kidney traits in FHS, while the lower part of the table contains the SNPs that were selected for their location in a candidate gene.

*Polymorphic nucleotides (alleles) are listed with respect to the (+) strand relative to the human reference sequence with the nucleotide in the reference sequence listed first; strand information refers to this reference sequence. **†**Trait lists: 1: all 3 traits, 2: CKD&eGFR, 3: cys&eGFR, 4: CKD, 5: eGFR, 6: cys. Statistical significance for replication was determined *a priori *at p = 0.00625 for SNPs selected based on p-value and 0.05 for SNPs selected as candidates. P-values shown for ARIC are derived from all participants not missing any covariates (n = 8,717), sample size varies slightly due to missing individual SNPs. Annotation based on USCS Genome Browser, assembly March 2006 (NCBI Build 36.1). Gene names in italics indicate the SNP is located in the gene, parentheses indicate proximity to a gene or mRNA, SNPs more than 400 kb away from the closest known gene or mRNA are defined as located in a gene desert. Abbreviations: MAF: minor allele frequency, GEE: generalized estimating equation, FBAT: family-based association test, GLM: generalized linear model, CKD: chronic kidney disease, cys: cystatin C, eGFR: estimated glomerular filtration rate, ns cSNP: non-synonymous coding SNP.

Among the SNPs selected for their location in a candidate gene, rs3779748 (*EYA1*) was significantly associated with CKD not at ARIC visit 4 but at visit 1 (OR 1.22 per each increase in T allele, p = 0.01) [see Additional file 3]. The same risk allele had been observed in FHS. However, since rs3779748 had been significantly associated with only the traits eGFR and cystatin C but not CKD in FHS, this does not constitute a true replication. Another SNP, rs10520688 in *IQGAP1*, was significantly associated with CKD at ARIC visit 4 (p = 0.02), but the risk allele was opposite from the one observed in FHS. None of the other SNPs selected for their location in a candidate gene was significantly associated with either kidney trait in ARIC.

### Secondary analyses

In secondary analyses, the association of all 16 SNPs was investigated prospectively with kidney disease progression in ARIC. Over a mean follow-up of 14.7 years, there were 836 white ARIC participants with kidney disease progression. None of the SNPs was significantly associated with kidney disease progression among white ARIC participants at the pre-specified levels of significance. However, the SNP rs6495446 (*MTHFS*), which replicated among white ARIC participants for the association with CKD, showed a hazard ratio of 1.13 per each increase in C allele for kidney disease progression (95% CI 1.01–1.26, p = 0.041). The proportional hazards assumption was met.

We further investigated the association of rs6495446 and CKD at ARIC visit 4 stratified by sex as well as by age (<60 years, ≥ 60 years). In these hypothesis-generating analyses, we observed stronger effects in men (OR = 1.32, 95% CI 1.11–1.57) compared to women (OR = 1.15, 95% CI 0.95–1.40; p-interaction = 0.33) and in participants aged <60 years (OR = 1.54, 95% CI 1.12–2.11) compared to those aged ≥ 60 years (OR = 1.16, 95% CI 1.01–1.34; p-interaction = 0.09).

**Figure 2:**
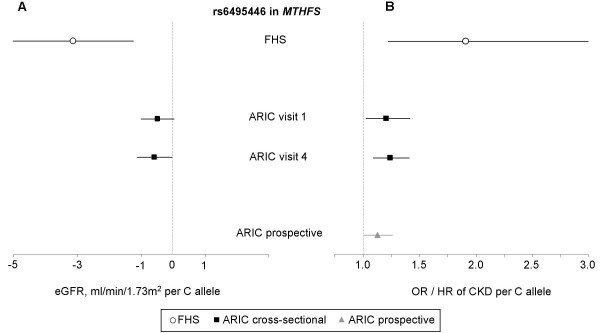
**Results from multivariable adjusted association analyses of rs6495446 in MTHFS and both eGFR (panel A) and CKD (panel B) in FHS and ARIC**. Risk estimates refer to each additional copy of the C allele. For ARIC, odds ratios of CKD per risk allele and the hazard ratio of kidney disease progression per risk allele are presented on the same scale. Horizontal bars represent 95% confidence intervals. Abbreviations: eGFR: estimated glomerular filtration rate, OR: odds ratio, CKD: chronic kidney disease.

For SNP rs6495446, which had replicated among the ARIC white sample, the FHS data were re-analyzed in secondary analyses using multivariable regression of the raw trait in order to parallel the analyses conducted in ARIC, thus allowing for a direct comparison of effect size estimates. Per each increase in C allele, the OR of CKD was 1.91 (95% CI 1.22–2.99, p = 0.005), and mean eGFR was lower by 3.1 ml/min/1.73 m^2 ^(95% CI 1.2–5.0, p = 0.001). Figure 2 shows these risk estimates in relation to the risk estimates obtained for eGFR (panel A) and CKD (panel B) in ARIC, as well as in relation to the risk estimates obtained from prospective analyses in ARIC.

We also conducted sensitivity analyses in the ARIC Study using an overall cutoff of eGFR <60 ml/min/1,73 m^2 ^to define CKD in order to evaluate the most commonly used clinical definition of CKD [18]. While the direction of the association of rs6495446 and CKD in ARIC whites at study visit 4 was consistent and the magnitude largely unchanged, the odds ratio was no longer statistically significant (OR 1.12, p = 0.075) as it had been using the sex-specific cutoffs.

## Discussion

Here we present results from the first complete GWAS of renal traits, comprised of results from FHS, the discovery sample (stage I) [14], and the large population-based ARIC Study, the replication sample (stage II). The association of SNP rs6495446 in *MTHFS *with CKD replicated in white ARIC participants at study visit 4. Another SNP, rs3779748 in *EYA1*, was significantly associated with CKD at study visit 1, a kidney trait different from but related to the ones significantly associated in FHS, eGFR and cystatin C. Our findings may provide guidance for investigators wishing to further replicate the associations from this GWAS of renal traits, as well as present considerations and mechanisms of SNP selection for replication studies.

We cannot compare our findings to existing literature, since this is the first study presenting replicated results from a GWAS of renal traits. Apart from the results in FHS, another study has published initial but not replicated association results from genome-wide tests for the traits serum creatinine, eGFR, and creatinine clearance in 2,000 white hypertensive individuals [33]. Findings on the association of specific SNPs with serum creatinine or eGFR were not presented as none of these associations met the significance threshold for further investigation of p < 10^-5 ^chosen by the authors [33].

SNP rs6495446, an intronic SNP in the gene *MTHFS *on chromosome 15q25.1, was significantly associated with CKD in white ARIC participants at study visit 4 (OR 1.24 per each C allele, 95% confidence interval 1.09–1.41, p = 0.001), replicating the trait and risk allele observed in the initial screen. Comparison of the effect sizes as well as the proportion of eGFR variance explained by rs6495446 between the FHS and white ARIC samples showed greater magnitude of the effects in FHS, consistent with the phenomenon of the "winner's curse": associations in the replication sample are often of lesser magnitude than in the initial sample, especially in samples with relatively low power to detect moderate effect sizes, because only the strongest associations in this initial sample were selected to be followed up. Further replication of the association of *MTHFS *rs6495446 and CKD in additional studies is warranted, particularly in light of the small effect size observed in the replication and the fact that the association reached statistical significance at the pre-specified level for one but not both traits and visits studied in ARIC.

The gene *MTHFS *codes for the enzyme methenyltetrahydrofolate synthetase, which is expressed in human and rat kidney and has been reported to play a role in folate turnover and accumulation [34]. To our knowledge, there have not been any prior reports linking this gene to renal traits or kidney disease, although folate supplementation is known to reduce homocysteine levels, which are commonly elevated in individuals with advanced kidney disease [35,36]. The SNP rs6495446 is located in intron 2 of *MTHFS*. The only coding SNP in *MTHFS *provided in the public database dbSNP is rs8923 in exon 3 (T202A); this SNP was not included on the genotyping chip. In the HapMap CEU sample, rs8923 and rs6495446 are grouped into one LD block (pair-wise D' = 1.0; r^2 ^= 0.38). Interestingly, when we searched a database containing results from a GWAS of global gene expression [37], the association of the rs6495446 C allele with higher expression levels of a *MTHFS *gene transcript was genome-wide significant (p = 3.3 × 10^-10^). The coding variant rs8923 was not genotyped in this study, but its location is flanked on both sides by the two markers showing the strongest association with *MTHFS *transcript levels out of all 408,273 SNPs investigated. rs6495446 by itself accounted for 13% of the total expression variance of this *MTHFS *transcript [38]. Although this observation does not allow for the conclusion that altered *MTHFS *mRNA levels associated with rs6495446 are causally related to kidney disease, it provides some functional evidence for rs6495446 or a variant in LD with it. Upcoming denser GWAS may help to further fine-map the observed association

Another SNP significantly associated with CKD in ARIC whites at visit 1, rs3779748, is located in the gene eyes absent homolog 1 (Drosophila) (*EYA1*) on chromosome 8q13.3. Mutations in *EYA1 *cause the Mendelian syndrome branchiootorenal dysplasia syndrome (MIM# 113650) featuring a renal phenotype ranging from mild renal hypoplasia to complete absence of the kidneys [25]. Although the same risk allele for rs3779748 was identified in both FHS and ARIC whites, this SNP was associated only with the traits eGFR and cystatin C in FHS. Although CKD and eGFR are highly correlated traits in ARIC, the observed association should therefore not be considered a true replication. Moreover, the association with CKD at ARIC visit 1 was no longer observed at ARIC visit 4, and we can therefore not exclude a chance finding.

Strengths of the work presented here include high power for replication of the association between common genetic variants and renal traits, as well as the availability of a replication study similar in design to the FHS Study, namely another community-based, prospective study with rigorously collected phenotype information. We were therefore able to adjust our analyses for the same covariates as FHS, leading to better comparability of the results across studies. Finally, we were able to prospectively evaluate the association with incident kidney disease progression in secondary analyses.

When interpreting the findings presented here, several limitations should be kept in mind. First, there are limitations to the original 100 K genome-wide screen for renal traits in FHS, which are discussed in detail elsewhere [13,14]. Among them is the limited power to discover moderate genetic effects of the size we observed (OR~1.2), especially for dichotomous but also for continuous traits. It may therefore be expected that future, better powered screens will identify additional variants of comparable effect size that could not be detected in the FHS 100 K screen. Further, the FBAT test is underpowered as it only relies on a subset of informative families. Therefore, FBAT results should not be interpreted with the same degree of confidence as GEE results. Despite these limitations, however, this genome-wide screen successfully identified a SNP in *CST3*, the gene encoding the cystatin C protein, as associated with serum cystatin C levels at genome-wide significance [14]. This increases confidence that the FHS 100 K genome-wide screen was able to identify associations which may represent true findings. Another potential limitation in the initial genome-wide screen was the use of a liberal call rate of 80%. We tried to address this in our replication study by using only those SNPs having call rates >90% in FHS. Moreover, SNPs with minor allele frequencies <10% were excluded from the initial association analyses in FHS, as an excess number of significant results was observed for SNPs with low minor allele frequencies [13]. We could therefore not investigate a putative contribution to the phenotypic variation by rare genetic variants, as has been reported for other complex genetic traits [39]. However, genome-wide association in general is not a good technique for detecting rare variants [40].

We further cannot exclude the possibility that undetected population stratification might have influenced our results, but prior investigations within FHS have found no evidence for the presence of population stratification [13]. In addition, the magnitude of the association between rs6495446 and CKD in ARIC did not differ significantly by ARIC Study center (p-interaction = 0.3).

Secondly, the selection of only 16 SNPs for further genotyping might not be sufficient for a comprehensive evaluation of all strongly associated SNPs from the FHS 100 K genome-wide association scan of renal traits. In particular, the selection and examination of a single SNP for a given genetic region may not be sufficient to capture the full effect of genetic variation in this region. Despite good power to detect significant associations between common SNPs and renal traits in ARIC, we observed true replication for only one of the selected SNPs, rs6495446. This may have been influenced by the selection strategy we used to prioritize SNPs for follow-up genotyping. A formal comparison between SNPs selected based on joint association with related phenotypes and those selected based on low p-values and biologic reasons is limited by the small number of selected SNPs, but our data does not lend strong support to either strategy. The results presented here, specifically for rs6495446 in *MTHFS*, should be further replicated and additional variants in *MTHFS *should be genotyped in future studies for fine-mapping of the association before a definite conclusion about the presence or absence of a moderate-sized association between variation in *MTHFS *and renal traits can be drawn.

Limitations to the phenotype definition include the lack of serum cystatin C measurements in ARIC as well as estimation of GFR. Although serum creatinine measurements were calibrated in both FHS and ARIC using the same method, GFR estimation based on serum creatinine has inherent limitations including lower accuracy in the higher GFR ranges [41]. We tried to address this issue by selecting SNPs for replication that were significantly associated with more than one renal trait in FHS, and in fact 50% of the SNPs selected for further genotyping in ARIC were associated with all 3 kidney traits investigated in FHS.

## Conclusion

In summary, the association of SNP rs6495446 in *MTHFS *with CKD was replicated in an independent study sample of white ARIC participants, constituting the first GWAS of kidney disease traits including both discovery and replication. Further work is needed to fully characterize the association of genetic variants in *MTHFS *with kidney disease. These findings highlight the importance of replication of initial GWAS findings to identify common SNPs associated with renal function traits.

## Competing interests

The authors declare that they have no competing interests.

## Authors' contributions

AK obtained funding, participated in conception and design of the study, selected SNPs for replication, performed the statistical analyses in the ARIC Study, and drafted the manuscript. WHLK was involved in ARIC funding and data collection, participated in the conception and design of the study and helped draft sections of the manuscript. S–JH assisted in the design of the study and in performing the statistical analyses for FHS. EB carried out genotyping for the ARIC Study and interpretation of study results. QY was involved in FHS study design and data analyses. DL contributed to the collection of data for FHS, interpretation of results, and critical edits to the manuscript. EJB participated in discussions of study design and provided critical revisions to the manuscript. MGL participated in the interpretation of results and revision of the manuscript. BCA was involved in ARIC funding, participated in the conception and design of the study and helped draft a section of the manuscript. JC was involved in ARIC funding and data collection, participated in the conception and design of the study and helped draft sections of the manuscript. CSF conceived of the study, helped acquire the data, interpret the findings, and helped to draft and critically revise the manuscript. All authors read and approved the final manuscript.

## Pre-publication history

The pre-publication history for this paper can be accessed here:



## Supplementary Material

Additional file 1 Word document, Supplementary Table 1: Study Characteristics in Black ARIC Participants. Lists sample characteristics for black ARIC participants at ARIC visits 1 and 4 analogous to Table 1. Click here for file

Additional file 2 Word document, Supplementary Table 2: Minor allele frequencies for all SNPs genotyped in 4,253 black ARIC participants. Lists SNP characteristics in black ARIC participants analogous to Table 2. Click here for file

Additional file 3 Word document, Supplementary Table 3: Replication data of 16 SNPs significantly associated with renal traits on the FHS 100 K chip in 11,217 white participants at ARIC visit 1. Lists association results between SNPs and renal traits in white ARIC participants at ARIC visit 1, analogous to Table 3. Click here for file
