# Supplementary material for: Genome-wide association study for renal traits in the Framingham Heart and Atherosclerosis Risk in Communities Studies
Source: BMC Med Genet. 2008 Jun 3;9:49. doi: 10.1186/1471-2350-9-49 (PMC2430944; doi:10.1186/1471-2350-9-49)
Supplement: Additional file 1 — Word document, Supplementary Table 1: Study Characteristics in Black ARIC Participants. Lists sample characteristics for black ARIC participants at ARIC visits 1 and 4 analogous to Table 1. [file 1471-2350-9-49-S1.doc]

**Supplementary Table 1: Study Characteristics in Black ARIC Participants**

|  | **ARIC blacks** | |
| --- | --- | --- |
|  | **visit 1** | **visit 4** |
| Characteristic |  |  |
| Sample size n | 3894 | 2358 |
| Age, years | 53.5 (5.8) | 61.8 (5.6) |
| Male, % | 38.6 | 35.8 |
| Systolic blood pressure, mmHg | 129 (21) | 133.5 (20) |
| Antihypertensive medication, % | 43.5 | 54.3 |
| Diabetes mellitus, % | 19.4 | 26.2 |
| Body mass index, kg/m^2^ | 29.6 (6.1) | 30.6 (6.3) |
| Current smokers, % | 29.9 | 17.3 |
| High density lipoprotein cholesterol, mg/dl | 54.9 (17.5) | 53.0 (16.8) |
| Serum creatinine, mg/dl | 0.93 (0.76) | 1.00 (0.59) |
| eGFR, ml/min/1.73m^2^ | 102.6 (25.0) | 89.5 (21.7) |
| Prevalent CKD*, n (%) | 127 (3.3) | 173 (7.3) |
| Incident CKD^†^, n | N/A | 568 |
| Serum cystatin C, mg/l | N/A | N/A |

Data are presented as mean (standard deviation) for continuous and proportion for categorical variables for individuals with information on all characteristics available at FHS Offspring exam 7and ARIC visits 1 and 4. *CKD was defined as eGFR <59 ml/min/1.73m^2^ (women) and <64 ml/min/1.73m^2^ (men). ^†^For definition of incident CKD and duration of follow-up see methods section. Abbreviations: eGFR: estimated glomerular filtration rate, CKD: chronic kidney disease.
