# Supplementary material for: Genome-wide association study for renal traits in the Framingham Heart and Atherosclerosis Risk in Communities Studies
Source: BMC Med Genet. 2008 Jun 3;9:49. doi: 10.1186/1471-2350-9-49 (PMC2430944; doi:10.1186/1471-2350-9-49)
Supplement: Additional file 2 — Word document, Supplementary Table 2: Minor allele frequencies for all SNPs genotyped in 4,253 black ARIC participants. Lists SNP characteristics in black ARIC participants analogous to Table 2. [file 1471-2350-9-49-S2.doc]

**Supplementary Table 2: Minor allele frequencies for all SNPs genotyped in 4,253 black ARIC participants**

|  |  |  | **n** | **MAF** |
| --- | --- | --- | --- | --- |
| **SNP** | **gene** | **location** | **ARIC black** | **ARIC black** |
|  |  |  |  |  |
| ***Selected for low p-value only*** | | |  |  |
| rs4553158 | *MIER1* | chr1:67148467 | 3,876 | 0.04 (G) |
| rs6831700 | *WDR19* | chr4:39079530 | 3,886 | 0.29 (T) |
| rs2419912 | (BC047601) | chr5:157777113 | 3,943 | 0.43 (T) |
| rs2228210 | *HIVEP1* | chr6:12230160 | 3,891 | 0.07 (G) |
| rs10509132 | *ANK3* | chr10:61995671 | 3,942 | 0.40 (T) |
| rs1613631 | *KRT84* | chr12:51062202 | 3,873 | 0.42 (T) |
| rs6495446 | *MTHFS* | chr15:77942037 | 3,934 | 0.43 (T) |
| rs2827732 | gene desert | chr21:23107838 | 3,892 | 0.27 (A) |
|  |  |  |  |  |
| ***Selected as a candidate*** | |  |  |  |
| rs2061063 | *FRAS1* | chr4:79591766 | 3,895 | 0.14 (G) |
| rs4835136 | *NR3C2* | chr4:149627601 | 3,909 | 0.20 (T) |
| rs1743955 | *SGK1* | chr6:134562589 | 3,836 | 0.40 (C) |
| rs4148686 | *CFTR* | chr7:116728468 | 3,926 | 0.28 (G) |
| rs3779748 | *EYA1* | chr8:72410728 | 3,897 | 0.27 (C) |
| rs1455177 | BC047388 | chr9:3782613 | 3,810 | 0.27 (C) |
| rs10520688 | *IQGAP1* | chr15:88752520 | 3,935 | 0.24 (G) |
| rs2839235 | *PCNT* | chr21:46625020 | 3,894 | 0.49 (T) |

The sample size n presented here is for all successfully genotyped individuals irrespective of whether all covariates for regression analyses were available. Abbreviations: SNPs: single nucleotide polymorphisms, n: sample size, MAF: minor allele frequency.
