# Supplementary material for: Genome-wide association study for renal traits in the Framingham Heart and Atherosclerosis Risk in Communities Studies
Source: BMC Med Genet. 2008 Jun 3;9:49. doi: 10.1186/1471-2350-9-49 (PMC2430944; doi:10.1186/1471-2350-9-49)
Supplement: Additional file 3 — Word document, Supplementary Table 3: Replication data of 16 SNPs significantly associated with renal traits on the FHS 100 K chip in 11,217 white participants at ARIC visit 1. Lists association results between SNPs and renal traits in white ARIC participants at ARIC visit 1, analogous to Table 3. [file 1471-2350-9-49-S3.doc]

**Supplementary Table 3: Replication data of 16 SNPs significantly associated with renal traits on the FHS 100K chip in 11,217 white participants at ARIC visit 1.** The upper part of the table contains the SNPs selected for replication based on a low p-value (<0.01) for association with all of the 3 kidney traits in FHS, while the lower part of the table contains the SNPs that were selected for their location in a candidate gene.

| **SNP** | **Gene** | **Strand** | **Alleles*** | **MAF** | | **FHS** | | | | **ARIC** |
| --- | --- | --- | --- | --- | --- | --- | --- | --- | --- | --- |
|  |  | **FHS / ARIC** |  | **FHS** | **ARIC** | **Trait**  **List†** | **Trait** | **GEE**  **p-value** | **FBAT**  **p-value** | **GLM**  **p-value** |
|  |  |  |  |  |  |  |  |  |  |  |
| ***Selected for low p-value only*** | | |  |  |  |  |  |  |  |  |
| rs4553158 | *MIER1* | -/+ | A/G | 0.13(C) | 0.17(G) | 1,2,3,4 | CKD | 4.3xe^-4^ | 0.739 | 0.369 |
|  |  |  |  |  |  |  | cys | 0.006 | 0.432 | -- |
|  |  |  |  |  |  |  | eGFR | 0.003 | 0.914 | 0.877 |
| rs6831700 | *WDR19* | -/+ | G/T | 0.34(C) | 0.33(G) | 1 | CKD | 0.004 | 0.001 | 0.601 |
|  |  |  |  |  |  |  | cys | 0.344 | 0.005 | -- |
|  |  |  |  |  |  |  | eGFR | 0.034 | 0.001 | 0.810 |
| rs2419912 | (BC047601) | +/+ | T/C | 0.49(C) | 0.46(C) | 1 | CKD | 0.002 | 0.144 | 0.883 |
|  |  |  |  |  |  |  | cys | 0.003 | 0.030 | -- |
|  |  |  |  |  |  |  | eGFR | 9.4xe^-5^ | 0.342 | 0.009 |
| rs2228210 | *HIVEP1* | +/+ | A/G | 0.29(G) | 0.36(G) | 1,2,3 | CKD | 0.017 | 0.007 | 0.873 |
| ns cSNP |  |  |  |  |  |  | cys | 0.003 | 0.027 | -- |
|  |  |  |  |  |  |  | eGFR | 2.3xe^-4^ | 0.005 | 0.850 |
| rs10509132 | *ANK3* | +/+ | G/T | 0.45(G) | 0.48(G) | 1,2,3 | CKD | 0.009 | 0.855 | 0.393 |
|  |  |  |  |  |  |  | cys | 0.002 | 0.002 | -- |
|  |  |  |  |  |  |  | eGFR | 0.002 | 0.031 | 0.614 |
| rs1613631 | *KRT84* | -/+ | T/G | 0.2(C) | 0.20(G) | 1,2,3 | CKD | 0.048 | 0.002 | 0.692 |
|  |  |  |  |  |  |  | cys | 0.177 | 0.009 | -- |
|  |  |  |  |  |  |  | eGFR | 0.013 | 0.003 | 0.813 |
| rs6495446 | *MTHFS* | +/+ | C/T | 0.24(T) | 0.27(T) | 1,2,3 | CKD | 0.003 | 0.429 | 0.024 |
|  |  |  |  |  |  |  | cys | 0.006 | 0.149 | -- |
|  |  |  |  |  |  |  | eGFR | 0.001 | 0.167 | 0.073 |
| rs2827732 | gene desert | -/+ | C/A | 0.19(T) | 0.16(A) | 1,2,3 | CKD | 0.001 | 0.004 | 0.962 |
|  |  |  |  |  |  |  | cys | 0.002 | 4.9x10^-4^ | -- |
|  |  |  |  |  |  |  | eGFR | 0.002 | 0.025 | 0.081 |
| ***Selected as a candidate*** | | |  |  |  |  |  |  |  |  |
| rs2061063 | *FRAS1* | -/- | G/C | 0.33(G) | 0.35(G) | 2 | CKD | 0.009 | 0.015 | 0.516 |
|  |  |  |  |  |  |  | cys | 0.402 | 0.790 | -- |
|  |  |  |  |  |  |  | eGFR | 0.009 | 0.018 | 0.194 |
| rs4835136 | *NR3C2* | -/+ | C/T | 0.36(A) | 0.37(T) | 2,5 | CKD | 0.003 | 0.002 | 0.100 |
|  |  |  |  |  |  |  | cys | 0.971 | 0.260 | -- |
|  |  |  |  |  |  |  | eGFR | 0.001 | 0.001 | 0.492 |
| rs1743955 | *SGK1* | -/+ | T/C | 0.42(A) | 0.39(T) | 3 | CKD | 0.707 | 0.459 | 0.102 |
|  |  |  |  |  |  |  | cys | 0.096 | 0.004 | -- |
|  |  |  |  |  |  |  | eGFR | 0.006 | 0.007 | 0.593 |
| rs4148686 | *CFTR* | -/+ | C/G | 0.17(C) | 0.20(G) | 4 | CKD | 1.1xe^-4^ | 0.209 | 0.339 |
|  |  |  |  |  |  |  | cys | 0.914 | 0.291 | -- |
|  |  |  |  |  |  |  | eGFR | 0.060 | 0.555 | 0.867 |
| rs3779748 | *EYA1* | +/+ | T/C | 0.32(C) | 0.33(C) | 3 | CKD | 0.128 | 0.826 | 0.010 |
|  |  |  |  |  |  |  | cys | 0.008 | 0.435 | -- |
|  |  |  |  |  |  |  | eGFR | 0.006 | 0.593 | 0.809 |
| rs1455177 | BC047388 | +/- | C/G | 0.45(C) | 0.45(G) | 6 | CKD | 0.113 | 0.494 | 0.229 |
|  | (GLIS3) |  |  |  |  |  | cys | 6.0xe^-4^ | 0.045 | -- |
|  |  |  |  |  |  |  | eGFR | 0.059 | 0.669 | 0.850 |
| rs10520688 | *IQGAP1* | +/- | T/C | 0.15(C) | 0.14(G) | 3 | CKD | 0.043 | 0.837 | 0.372 |
|  |  |  |  |  |  |  | cys | 0.004 | 0.608 | -- |
|  |  |  |  |  |  |  | eGFR | 0.003 | 0.82 | 0.796 |
| rs2839235 | *PCNT* | +/+ | T/C | 0.14(C) | 0.13(C) | 3,5 | CKD | 0.028 | 0.134 | 0.846 |
|  |  |  |  |  |  |  | cys | 0.016 | 0.006 | -- |
|  |  |  |  |  |  |  | eGFR | 1.6xe^-5^ | 0.055 | 0.845 |

* Polymorphic nucleotides (alleles) are listed with respect to the (+) strand relative to the human reference sequence with the nucleotide in the reference sequence listed first; strand information refers to this reference sequence. **†**Trait lists: 1: all 3 traits, 2: CKD&eGFR, 3: cys&eGFR, 4: CKD, 5: eGFR, 6: cys. Statistical significance for replication was determined *a priori* at p=0.00625 for SNPs selected based on p-value and 0.05 for SNPs selected as candidates. P-values shown for ARIC are derived from all participants not missing any covariates (n=11,217), sample size varies slightly due to missing individual SNPs. Annotation based on USCS Genome Browser, assembly March 2006 (NCBI Build 36.1). Gene names in italics indicate the SNP is located in the gene, parentheses indicate proximity to a gene or mRNA, SNPs more than 400 kb away from the closest known gene or mRNA are defined as located in a gene desert. Abbreviations: MAF: minor allele frequency, GEE: generalized estimating equation, FBAT: family-based association test, GLM: generalized linear model, CKD: chronic kidney disease, cys: cystatin C, eGFR: estimated glomerular filtration rate, ns cSNP: non-synonymous coding SNP.
